# Observational Evaluation of Muscle Echointensity and EMG Insertional Activity in Poststroke Spasticity

**DOI:** 10.1002/mus.70186

**Published:** 2026-02-18

**Authors:** Rajiv Reebye, Ève Boissonnault, April Jeon, Mirko Filippetti, Gina McKernan, Oluwasanmi O. Adenaiye, Alessandro Picelli, Michael C. Munin

**Affiliations:** ^1^ Division of Physical Medicine and Rehabilitation University of British Columbia Vancouver Canada; ^2^ Canadian Advances in Neuro‐Orthopedics for Spasticity Consortium (CANOSC) Kingston Canada; ^3^ Faculty of Medicine and Dentistry, Division on Physical Medicine and Rehabilitation, Department of Medicine University of Alberta Edmonton Canada; ^4^ Department of Physical Medicine and Rehabilitation University of Pittsburgh School of Medicine Pittsburgh Pennsylvania USA; ^5^ Physical and Rehabilitation Medicine Section, Department of Neurosciences, Biomedicine and Movement Sciences University of Verona Verona Italy

**Keywords:** echointensity, electromyography, Modified Heckmatt Scale, muscle spasticity, ultrasonography

## Abstract

**Introduction/Aims:**

When spasticity occurs after a stroke, peripheral changes in spastic muscle architecture may develop. The primary objective was to determine if an association exists between spastic muscle echointensity (EI) measured by the Modified Heckmatt Scale (MHS) and changes in insertional activity detected by electromyography (EMG). The secondary aim was to investigate whether these changes are due to the effects of botulinum neurotoxin (BoNT).

**Methods:**

A total of 55 patients with poststroke spasticity were enrolled from three outpatient spasticity clinics. Muscle EI and needle EMG insertional activity were assessed for 8 muscles in each subject. Chi‐square tests or Fisher's exact tests for categorical variables were used to evaluate the association between muscle EI and EMG insertional activity, as well as the association between BoNT exposure and muscle changes.

**Results:**

For MHS Grade 1–2 muscles, 91.7% had normal insertional activity compared to 46.4% of MHS Grade 3–4 muscles (*p* < 0.001). In muscles with abnormal insertional activity, reduced or absent insertional activity was seen in 67.3% of MHS 3–4 muscles compared to only 16.7% for MHS 1–2 muscles, while increased insertional activity was seen in 32.7% of MHS 3–4 muscles and 83.3% of MHS graded 1–2 muscles. Exposure to BoNT did not impact the observed association between MHS and abnormal EMG insertional activity.

**Discussion:**

Increased EI is associated with abnormal EMG insertional activity. Exposure to BoNT does not explain the observed EI and EMG changes. Further research is needed to elucidate the significance and causes of muscle architectural and electromyographical transformation in poststroke spasticity.

**Abbreviations**
BoNT: botulinum neurotoxin
EI: echointensity
EMG: electromyography
MAS: Modified Ashworth Scale
MHS: Modified Heckmatt Scale
MRC: Medical Research Council scale for muscle strength
SD: standard deviation

## 1 Introduction

Spasticity is a sensorimotor disorder characterized by intermittent or sustained involuntary muscle activation and is a common and potentially problematic consequence of upper motor neuron disorders [1]. Central nervous system (CNS) damage can contribute to neural and nonneural changes. The neural phase involves CNS plastic rearrangements, including supraspinal and spinal rearrangements, leading to muscle overactivity. The nonneural changes involve immobilization of the tendon and muscle in the shortened position, leading to soft tissue plastic rearrangements and contractures that play crucial roles in the acute and delayed phases of spasticity [2, 3, 4, 5]. Histological and imaging methods demonstrate that spasticity and chronic disuse are associated with greater variability in muscle fiber size, proliferation of disorganized extracellular matrix, increased fat content, muscle shortening, atrophy, and sarcopenia [2, 3, 6]. By altering the histopathological properties of muscle, spasticity may also alter sonoacoustic features as evidenced by the direct relationship between increased echointensity (EI) and the severity of spasticity [7, 8]. Response to spasticity treatment, including botulinum neurotoxin (BoNT) injections, has been shown to be less in muscles with higher EI [9, 10, 11].

EI can be measured clinically using the Modified Heckmatt Scale (MHS), a semiquantitative tool that was validated in patients with spasticity against objective grayscale image analysis [12]. The MHS can determine if a spastic muscle appears normal or abnormal without relying on pixel intensity calculation or specialized image processing [12]. The scale had good to excellent inter‐rater reliability and good intra‐rater reliability [12]. Unlike the Classic Heckmatt Scale, which assesses homogeneous muscle changes seen in myopathies, the MHS accounts for the heterogeneous characteristic of spastic muscle [13]. However, the physiological significance of the different MHS scores has not been fully elucidated.

Patients with poststroke spasticity may show abnormal insertional activity on needle electromyography (EMG). In the subacute phase, increased insertional activity with fibrillation potentials and positive sharp waves can be observed even when there is no peripheral nerve lesion. This abnormal activity is believed to be related to the severity of hemiparesis and is likely due to transsynaptic degeneration, where the loss of upper motor neuron input leads to secondary changes in the lower motor neuron and muscle fibers [14, 15]. Typically, this increased insertional activity resolves within 6–12 months after the stroke [14, 15, 16].

Spastic muscles in patients with stroke may show reduced or absent insertional activity. This reduction occurs as muscle tissue is gradually replaced by fibrous connective tissue and fat [17]. The underlying mechanism is unknown but involves chronic disuse, impaired neural input, and ongoing muscle atrophy, which promote the replacement of contractile muscle fibers with noncontractile tissue [18].

The purpose of this study was to determine if an association exists in spastic poststroke muscles between abnormal EI assessed by validated clinician‐rated US measurements and insertional activity detected by needle EMG. The secondary aim was to investigate whether BoNT exposure can explain any relationship between US measurements and insertional activity. A better understanding of spastic muscle health after stroke could eventually inform treatment and support clinical decisions and research.

## 2 Methods

This was a cross‐sectional, multinational study (one American center [Pittsburgh], one Canadian center [Vancouver], one Italian center [Verona]). We recruited patients with poststroke spasticity from three outpatient clinics that were providing BoNT injections for muscle spasticity. Eight muscles in the affected hemibody were studied in each subject, regardless of whether or not the muscles were subsequently injected with botulinum toxin.

Inclusion criteria were age greater than 18 years and a diagnosis of ischemic or hemorrhagic stroke with associated spasticity affecting the upper and/or lower extremities. Included patients were either treated or being considered for treatment with BoNT injections for muscle spasticity. All muscles considered for BoNT injections had a Modified Ashworth Scale (MAS) [19] score between 1+ and 3. Exclusion criteria were as follows: major trauma or surgery in the affected limb that would affect muscle architecture, inability to assume a body position for proper image acquisition, hardware in the affected limb, previous surgical treatment for spasticity in the affected limb, and other neurologic or orthopedic conditions that could impact the affected limb.

Ethics approval was obtained from the University of Pittsburgh (United States; IRB # STUDY21120128), the University of British Columbia (Canada; IRB # H22‐00275), and the University of Verona (Italy; IRB # N251‐140912). Informed written consent was obtained from patients or caregivers at each site.

### 2.1 Demographic Data

Demographic data included age, sex, height, weight, hand dominance, city/country, diagnosis leading to spasticity (i.e., type of stroke), date of stroke, spasticity location, past medical history, medications, previous spasticity treatments (including BoNT and phenol neurolysis), physiotherapy and occupational therapy treatments, and adjunctive treatments. If a patient received prior BoNT injections, then the number of treatments, dates of treatments, muscles injected, type of BoNT, and dose were recorded. The same units were used for incobotulinumtoxinA and onabotulinumtoxinA. AbobotulinumtoxinA units were converted by dividing the dose by 2.5 so that all BoNT types were in equivalent units for this analysis.

### 2.2 Ultrasound Image Acquisition

Ultrasound machine settings were kept consistent at all three sites (Pittsburgh, Sonosite PX, Bothell, WA, USA; Vancouver, Esaote MyLab 7, Genoa, Italy; Verona, Esaote MyLab 70 XVision, Genoa, Italy). The examiners performed B‐mode ultrasonography with a linear transducer (scanning frequency 6–15 MHz) to evaluate muscles in the spastic limb. Examinations were performed by physicians with training in obtaining these images. The dynamic range setting was the same for all patients. Standardized locations for probe placement and standardized body positioning were used to obtain static ultrasound images of each muscle (see Table S1). A bony window was captured, if possible, to allow for comparison between muscle and bone echo. Investigators attempted to use the same maximum depth up to 5 cm. This, of course, could vary based on muscle atrophy, subcutaneous tissue, and body habitus. The following muscles were assessed in the spastic upper limb: biceps brachii, brachialis, flexor carpi radialis, pronator teres, and flexor digitorum superficialis. The following muscles were assessed in the spastic lower limb: rectus femoris, medial gastrocnemius, and lateral gastrocnemius. A brief virtual training session was conducted across the three sites before the study to optimize the uniformity of ultrasound image acquisition.

### 2.3 MHS

Muscle EI was visually evaluated in the transverse plane using the MHS grading scheme:

Grade 1: Normal echogenicity in more than 90% of the muscle that is distinct from bone echo.

Grade 2: Increased muscle echogenicity in 10%–50% of tissue, but with distinct bone echo and areas of normal muscle echo.

Grade 3: Marked increase in muscle echogenicity that affects 50%–90% of tissue with reduced distinction of bone echo from muscle.

Grade 4: Very strong muscle echogenicity with near complete loss of distinct bone echo from muscle in > 90% of tissue.

### 2.4 Needle EMG Insertional Activity

Needle EMG insertional activity was assessed using a monopolar injectable 37 or 50 mm electrode based on body habitus. Surface reference and ground electrodes were applied along with standard EMG filters. Each site had an EMG machine (Pittsburgh, Xltek Neuromax 1002ce, Gort, County Galway, Ireland), (Vancouver, Natus Ultrapro S100, Gort, County Galway, Ireland), (Verona, Nemus1 EBneuro, EB Neuro, Florence, Italy) to obtain muscle insertional activity. Under ultrasound guidance, two to three passes of the EMG electrode at a location determined by the MHS grade were completed in each muscle to assess insertional activity. While a few pockets of normal EI can be present in MHS 3–4 graded muscles, we assessed insertional activity in the largest area of muscle that determined the MHS grade to obtain a representative sample. The examiners were board‐certified in physical medicine and rehabilitation and trained in EMG. Insertional activity was graded into one of four separate and nonhierarchical categories:

1. No electrical activity, defined as absent muscle fiber potentials with needle insertion.
2. Reduced insertional activity, defined as a decrease in brief bursts of electrical activity with needle insertion.
3. Normal activity is noted with a burst of muscle fiber potentials lasting no longer than 300 ms.
4. Increased insertional activity characterized by fibrillation and positive wave potentials that persist > 300 ms.

Only the Pittsburgh site conducted EMG analyses on muscles classified as MHS Grades 1 and 2 to achieve a more balanced representation between the MHS 1–2 and MHS 3–4 groups.

### 2.5 Statistical Analysis

Descriptive statistics were used to summarize demographic and clinical characteristics, including means, standard deviations (SDs), interquartile ranges, medians, and ranges for continuous variables, as well as frequencies and percentages for categorical variables. MHS Grade 1–2 and 3–4 were combined to improve statistical power, resulting in greater precision of results and simplifying the interpretation of findings. Due to the small proportion of Grades 1 and 4, this approach provides a more parsimonious model and increased sample size. Similarly, due to the unbalanced proportions within EMG insertional activity, we collapsed the categories so that they could be classified as either normal or abnormal. To assess the relationships between EI as measured by the MHS and EMG insertional activity, we used chi‐square tests or Fisher's exact tests for categorical variables to evaluate the association between EMG insertional activity and muscle quality. Differences in EMG insertional activity across MHS grades were evaluated using the chi‐square test for categorical outcomes. For continuous variables, depending on the distribution, either *t*‐tests or Mann–Whitney *U* tests were conducted.

Among patients who had previously received BoNT treatment, not all muscles were necessarily injected based on clinical opinion. For the purposes of statistical analysis, muscles that never received BoNT injections were classified as naive regardless of whether the patient had received BoNT injections in other muscles. Both MHS and EMG insertional activity were evaluated for these untreated (naive) muscles.

For continuous variables such as age and body mass index, comparisons between groups were performed using *t*‐tests or nonparametric equivalents as appropriate. However, certain analyses, such as the relationship between the specific BoNT dose for individual muscles and MHS grade, were limited by sample size and variability, precluding formal statistical testing. Data categorized as “not reported” were not included in the statistical analyses.

The significance threshold was set at *p* < 0.05. All statistical analyses were two‐tailed and were conducted using R version 4.2.2. All analyses were performed in R version 4.5.0, developed and maintained by the R Core Team and distributed by the R Foundation for Statistical Computing (Vienna, Austria; https://www.r‐project.org).

## 3 Results

A total of 56 adults were recruited across three sites, with each participant enrolled at a single site. Detailed demographics for the entire cohort without statistical analysis are listed in Table 1. No participants received previous phenol injections. Ten participants had never received BoNT injections, including during this study.

**TABLE 1:** Baseline characteristics of study participants (*N* = 56).

Characteristic	Overall, *N* = 56	BoNT‐exposed patients, *N* = 46	BoNT‐naive patients, *N* = 10
Age (years), median (Q1, Q3)	61 (52, 70)	63 (53, 70)	57 (47, 64)
Sex, *n* (%)			
Female	27 (48.2%)	22 (47.8%)	5 (50.0%)
Male	29 (51.8%)	24 (52.2%)	5 (50.0%)
Body mass index (kg/m^2^), median (Q1, Q3)	26 (23, 29)	26 (23, 29)	27 (25, 29)
Not reported	1	1	0
Stroke type, *n* (%)			
Hemorrhagic stroke	16 (31.4%)	13 (31.0%)	3 (33.3%)
Ischemic stroke	35 (68.6%)	29 (69.0%)	6 (66.7%)
Not reported	5	4	1
Time since stroke (days), median (Q1, Q3)	2852 (1425, 5007)	3146 (1562, 5007)	1765 (919, 3609)
Affected side, *n* (%)			
Left	26 (46.4%)	22 (47.8%)	4 (40.0%)
Right	30 (53.6%)	24 (52.2%)	6 (60.0%)
Spasticity location, *n* (%)			
Both	42 (76.4%)	34 (75.6%)	8 (80.0%)
Lower limb	6 (10.9%)	5 (11.1%)	1 (10.0%)
Upper limb	7 (12.7%)	6 (13.3%)	1 (10.0%)
Not reported	1	1	0
Total lifetime BoNT sessions per patient, median (Q1, Q3)	9 (3, 15)	10 (6, 17)	0 (0, 0)
Cumulative lifetime BoNT dose per patient (equiv. units), median (Q1, Q3)	1858 (313, 4185)	2520 (1080, 4650)	0 (0, 0)
Statin use, *n* (%)	28 (50.9%)	25 (54.3%)	3 (33.3%)
Not reported	1	0	1
Diabetic medication use, *n* (%)	11 (20.0%)	10 (21.7%)	1 (11.1%)
Not Reported	1	0	1
Anticoagulation use, *n* (%)	15 (27.3%)	11 (23.9%)	4 (44.4%)
Not reported	1	0	1
Patient recruitment by site, *n* (%)			
Pittsburgh	20 (35.7%)	18 (39.1%)	2 (20.0%)
Vancouver	15 (26.8%)	13 (28.3%)	2 (20.0%)
Verona	21 (37.5%)	15 (32.6%)	6 (60.0%)

*Note:* BoNT‐naive subjects did not receive neurotoxin in any muscle.

Abbreviations: BoNT, botulinum neurotoxin; equiv. units, equivalent units; *N*, number of patients; Q1, first quartile; Q3, third quartile.

Table S2 shows the mean muscle grade by muscle group using the Medical Research Council (MRC) grade and BoNT exposure for the stroke‐affected side. The pattern of weakness is similar between BoNT‐treated and naive subjects, but formal statistical testing was not performed.

The relationship between MHS grade and EMG insertional activity across all 242 muscles was assessed. For MHS 1–2 muscles, 8.3% had abnormal insertional activity, which significantly differed from MHS 3–4 muscles, where 53.6% had abnormal insertional activity (*p* < 0.001). In muscles with abnormal insertional activity, reduced or absent insertional activity was seen in 67.3% of MHS 3–4 muscles compared to only 16.7% for MHS Grade 1–2 muscles, while increased insertional activity was seen in only 32.7% of MHS 3–4 muscles compared to 83.3% of MHS Grade 1–2 muscles. Table 2 describes the categories of abnormal EMG insertional activity that were detected in MHS 1–2 versus MHS 3–4 muscles regardless of BoNT exposure. Of note, MHS 3–4 muscles displayed a significantly higher percentage of reduced and absent insertional activity compared to MHS 1–2 muscles that primarily showed increased insertional activity, likely from the effects of chemodenervation. Table 3 reports the distribution of MHS grades and EMG insertional activity according to prior exposure to BoNT in individual muscles. Muscles exposed to BoNT had significantly more abnormal insertional activity in MHS 3–4 compared to MHS 1–2 graded muscles. This statistically significant relationship was also seen in BoNT‐naive muscles to the same degree.

**TABLE 2:** Abnormal EMG insertional activity categorized by MHS grade for all muscles regardless of BoNT exposure.

	MHS 1–2 (*n* = 12)	MHS 3–4 (*n* = 52)	Overall (*n* = 64)	*p*
Abnormal insertional activity, *n* (%)				
Fibrillation and positive wave potentials	10 (83.3%)	17 (32.7%)	27 (42.2%)	0.049
Reduced insertional activity	2 (16.7%)	29 (55.8%)	31 (48.4%)	
Absent insertional activity	0 (0.0%)	6 (11.5%)	6 (9.4%)	

Abbreviations: EMG, electromyography; MHS, Modified Heckmatt Scale; *n*, number of patients.

**TABLE 3:** MHS grade versus EMG insertional activity stratified by muscle BoNT exposure.

	BoNT‐exposed muscle			BoNT‐naive muscle		
	MHS 1–2 *N* = 85	MHS 3–4 *N* = 68	*p*	MHS 1–2 *N* = 60	MHS 3–4 *N* = 29	*p*
EMG insertional activity, *n* (%)			< 0.001			< 0.001
Normal	76 (89.4)	32 (47.1)		57 (95.0)	13 (44.8)	
Abnormal	9 (10.6)	36 (52.9)		3 (5.0)	16 (55.2)	

Abbreviations: BoNT, botulinum neurotoxin; EMG, electromyography; MHS, Modified Heckmatt Scale; *n*, number of patients.

Tables 4 and 5 show the five upper limb muscles and three lower limb muscles that were assessed for each subject and dichotomize each muscle as BoNT‐treated or naive. Each muscle then reports combinations of MHS grade (1–2 or 3–4) and EMG insertional activity (normal or abnormal). Only the brachialis showed a statistical difference between BoNT‐exposed versus naive muscles in terms of MHS grade and EMG activity.

**TABLE 4:** Five study upper limb muscles evaluated with the MHS and EMG insertional activity that were stratified by prior muscle BoNT exposure.

Characteristic	Overall	BoNT‐exposed muscle	BoNT‐naive muscle	*p*
Biceps brachii				
MHS grade/EMG activity, *n* (%)				0.791
MHS 1–2 and normal EMG	19 (73.1)	9 (75.0)	10 (71.4)	
MHS 1–2 and abnormal EMG	2 (7.7)	1 (8.3)	1 (7.1)	
MHS 3–4 and normal EMG	4 (15.4)	1 (8.3)	3 (21.4)	
MHS 3–4 and abnormal EMG	1 (3.8)	1 (8.3)	0 (0.0)	
Brachialis				
MHS grade/EMG activity, *n* (%)				0.015
MHS 1–2 and normal EMG	20 (64.5)	8 (44.4)	12 (92.3)	
MHS 1–2 and abnormal EMG	2 (6.5)	2 (11.1)	0 (0.0)	
MHS 3–4 and normal EMG	3 (9.7)	2 (11.1)	1 (7.7)	
MHS 3–4 and abnormal EMG	6 (19.3)	6 (33.3)	0 (0.0)	
Pronator teres				
MHS grade/EMG activity, *n* (%)				0.413
MHS 1–2 and normal EMG	14 (56.0)	7 (53.8)	7 (58.3)	
MHS 1–2 and abnormal EMG	0 (0.0)	0 (0.0)	0 (0.0)	
MHS 3–4 and normal EMG	4 (16.0)	1 (7.7)	3 (25.0)	
MHS 3–4 and abnormal EMG	7 (28.0)	5 (38.5)	2 (16.7)	
Flexor carpi radialis				
MHS grade/EMG activity, *n* (%)				> 0.999
MHS 1–2 and normal EMG	20 (76.9)	10 (71.4)	10 (83.3)	
MHS 1–2 and abnormal EMG	1 (3.8)	1 (7.1)	0 (0.0)	
MHS 3–4 and normal EMG	4 (15.3)	2 (14.3)	2 (16.7)	
MHS 3–4 and abnormal EMG	1 (3.8)	1 (7.1)	0 (0.0)	
Flexor digitorum superficialis				
MHS grade/EMG activity, *n* (%)				> 0.999
MHS 1–2 and normal EMG	16 (55.2)	8 (53.3)	8 (57.1)	
MHS 1–2 and abnormal EMG	5 (17.2)	3 (20.0)	2 (14.3)	
MHS 3–4 and normal EMG	3 (10.3)	2 (13.3)	1 (7.1)	
MHS 3–4 and abnormal EMG	5 (17.2)	2 (13.3)	3 (21.4)	

Abbreviations: BoNT, botulinum neurotoxin; EMG, electromyography; MHS, Modified Heckmatt Scale; *n*, number of patients.

**TABLE 5:** Three study lower limb muscles evaluated with the MHS and EMG insertional activity that were stratified by prior muscle BoNT exposure.

Characteristic	Overall	BoNT‐exposed muscle	BoNT‐naive muscle	*p*
Lateral gastrocnemius				
MHS grade/EMG activity, *n* (%)				0.426
MHS 1–2 and normal EMG	16 (37.2)	11 (31)	5 (62.5)	
MHS 1–2 and abnormal EMG	1 (2.3)	1 (2.9)	0 (0.0)	
MHS 3–4 and normal EMG	13 (30.2)	12 (34)	1 (12.5)	
MHS 3–4 and abnormal EMG	13 (30.2)	11 (31)	2 (25.0)	
Medial gastrocnemius				
MHS grade/EMG activity, *n* (%)				0.153
MHS 1–2 and normal EMG	11 (28.9)	8 (30.8)	3 (25.0)	
MHS 1–2 and abnormal EMG	1 (2.6)	1 (3.8)	0 (0.0)	
MHS 3–4 and normal EMG	10 (26.3)	9 (34.6)	1 (8.3)	
MHS 3–4 and abnormal EMG	16 (42.1)	8 (30.8)	8 (66.7)	
Rectus femoris				
MHS grade/EMG activity, *n* (%)				0.328
MHS 1–2 and normal EMG	17 (70.8)	15 (75.0)	2 (50.0)	
MHS 1–2 and abnormal EMG	0 (0.0)	0 (0.0)	0 (0.0)	
MHS 3–4 and normal EMG	4 (16.7)	3 (15.0)	1 (25.0)	
MHS 3–4 and abnormal EMG	3 (12.5)	2 (10.0)	1 (25.0)	

Abbreviations: BoNT, botulinum neurotoxin; EMG, electromyography; MHS, Modified Heckmatt Scale; *n*, number of patients.

Table S3 shows the lifetime BoNT injection history by muscle for BoNT‐exposed muscles only.

## 4 Discussion

The primary finding of this study is a direct association between higher MHS grades and abnormal EMG insertional activity in poststroke spasticity patients. Specifically, muscles classified as MHS Grades 3–4 exhibited a significantly greater frequency of abnormal insertional activity compared to MHS Grades 1–2 muscles, a relationship that was consistent across all 242 muscles analyzed. MHS 3–4 muscles displayed a significantly higher percentage of reduced or absent insertional activity than MHS 1–2 muscles, which primarily showed increased insertional activity likely due to chemodenervation. Importantly, this association held true regardless of whether the muscles had prior exposure to BoNT treatment. These results suggest that abnormal EMG findings in spastic muscles with higher MHS grades are not solely due to BoNT exposure, but rather reflect underlying muscle pathology such as edema, fibrosis, fatty infiltration, or thickened epimysium or perimysium [20, 21, 22].

Although several papers described BoNT injections contributing to muscle atrophy, increased collagen, and shifts in myosin‐heavy chains on histological slides of animal subjects [20, 22], our data demonstrate that BoNT exposure alone does not account for the observed differences in MHS grades and EMG activity. This finding is consistent with previous literature suggesting that BoNT has no cytotoxic effect and does not increase EI [21, 23, 24]. The fact that both groups show similar relationships indicates that factors beyond BoNT are influencing changes in muscle properties. A statistically significant difference between BoNT‐exposed and naive muscles was observed only in the brachialis. We suspect that this may be attributed to the limited sample size in the MHS 3–4 group. The lack of statistically significant differences in the remaining seven muscles further supports the idea that BoNT is not the primary driver of these changes.

Several factors may explain the underlying phenomena responsible for higher MHS grades and EMG abnormalities in spastic muscles. Literature suggests that phenomena involving fibrosis, fibrofatty infiltration, and thickening of connective tissue occur after a stroke [25, 26]. Disease duration, severity of the paresis and of the spasticity, functional impairment, and pathophysiologic phenomena such as sarcopenia also likely contribute to progressive muscle transformation over time [5, 7, 14, 23, 27, 28, 29, 30, 31]. Because all subjects experienced their strokes more than a year ago, transsynaptic degeneration is an unlikely explanation for the observed active denervation [16]. Additional genetic and epigenetic factors could also predispose to the development of poststroke soft tissue transformation [32]. Further study is therefore required to determine the significance of increased MHS grades, EMG abnormalities, and their potential causes.

Our data suggests that using the MHS and EMG together can help to better understand the quality of poststroke spastic muscles to optimize BoNT treatment. In muscles with MHS 3–4 and abnormal insertional activity, BoNT injections may not be effective. This new hypothesis will require further study to be confirmed or refuted. This study does not imply that it is mandatory to use both needle EMG and ultrasound together for BoNT injections, or that guidance with one modality is better than the other. Literature in neuromuscular disorders suggests that muscle ultrasound and needle EMG assess different aspects of muscle pathology [33]. Similarly, we hypothesize that EMG and ultrasound can be used in a complementary fashion to identify distinct features of spastic muscle.

## 5 Limitations

EMG evaluation for MHS 1–2 muscles was conducted only in one site (Pittsburgh). We do acknowledge that this strategy may induce a sampling bias. There was heterogeneity across the three sites because patients received intramuscular BoNT injections with both the dose and muscles chosen individually by their treating physician, rather than following a standardized injection protocol. The BoNT‐naive sample may be enriched with fewer spastic muscles, potentially masking any effect of BoNT in producing sonographic or electromyographic abnormalities.

Some muscles assessed by ultrasound did not receive BoNT injections, leading to unequal group sizes and potentially reduced statistical power for certain comparisons. Fewer than 70 BoNT‐exposed and 30 BoNT‐naive muscles had an MHS Grade of 3–4. In addition, the cross‐sectional design of this study did not allow for assessment of time‐dependent changes; longitudinal follow‐up is required to elucidate the progression of EI and EMG insertional activity over time. Finally, as investigators were not blinded to participants' clinical status or BoNT dosing, the potential for assessment bias cannot be excluded.

## 6 Conclusion

Our findings indicate that in poststroke patients, the combination of MHS grade and EMG insertional activity reveals key changes in spastic muscle, which are likely influenced by a range of factors such as connective tissue thickening, chronic denervation, disease duration, functional impairment, sarcopenia, and spasticity severity. Our data support that BoNT alone does not account for the observed differences, emphasizing the need for further research to clarify the individual contributions of these factors and to refine strategies for optimizing BoNT utilization.

## Author Contributions

R.R., È.B., and M.C.M. contributed to conceptualization, data curation, formal analysis, funding acquisition, investigation, methodology, writing – original draft, and writing – review and editing. A.J. contributed to methodology, project administration, writing – original draft, and writing – review and editing. G.M. and O.O.A. contributed to data curation, formal analysis, software, writing – original draft, and writing – review and editing. M.F. and A.P. contributed to conceptualization, investigation, writing – original draft, and writing – review and editing.

## Funding

This work was supported by the Canadian Advances in Neuro‐Orthopedics for Spasticity Consortium and the National Institutes of Health through the Clinical and Translational Science Institute (CTSI) at the University of Pittsburgh (UL1‐TR‐001857).

## Ethics Statement

We confirm that we have read the Journal's position on issues involved in ethical publication and affirm that this report is consistent with those guidelines.

## Conflicts of Interest

Rajiv Reebye has received honorarium for lectures and symposia from AbbVie, Merz, and Ipsen. Ève Boissonnault has received honorarium for lectures from AbbVie, Merz, Ipsen, and Pacira, advisor activity from Merz and Ipsen, consultant services from Pacira, educational grant from AbbVie, and research support from AbbVie and Pacira. Alessandro Picelli has received honorarium for lectures, symposia, and advisory activity from AbbVie, Merz, and Ipsen. Mirko Filippetti has received honorarium for advisor activity from AbbVie, as well as scientific conference sponsorships from AbbVie and Ipsen. Michael C. Munin has received research support from AbbVie, Merz, and Pacira, as well as honorarium from AbbVie, Revance, and Merz. The other authors declare no conflicts of interest.

## Supporting information


**Table S1:** Guidelines for obtaining echointensity ratings by muscle.
**Table S2:** Mean muscle strength (MRC 0–5) by muscle group and BoNT exposure.
**Table S3:** Lifetime BoNT history by muscle (treated muscles only).

## Data Availability

The data that support the findings of this study are available from the corresponding author upon reasonable request.
