# Supplementary material for: Observational Evaluation of Muscle Echointensity and EMG Insertional Activity in Poststroke Spasticity
Source: Muscle Nerve. 2026 Feb 18;73(5):858–66. doi: 10.1002/mus.70186 (PMC13047936; doi:10.1002/mus.70186)
Supplement: Supplementary file 1 — Table S1: Guidelines for obtaining echointensity ratings by muscle. Table S2: Mean muscle strength (MRC 0–5) by muscle group and BoNT exposure. Table S3: Lifetime BoNT history by muscle (treated muscles only). [file MUS-73-858-s001.docx]

**Supplemental Table 1: Guidelines for obtaining echointensity ratings by muscle**

| **Selected muscles** | **Scanning instructions** |
| --- | --- |
| **Upper limb muscles** |  |
| Biceps Brachii | Supine as much as possible with forearm extended.  Distal 2/3 of arm. Choose medial or lateral head. |
| Brachialis | Supine as much as possible and forearm extended.  Very distal/lateral.  Biceps sits superficial to brachialis and should be out of view.  Brachialis posterior/lateral to biceps. |
| Flexor Carpi Radialis | Supine as much as possible and forearm extended.  Image upper third of forearm.  Attempt to see two heads of PT with median nerve inferior and FCR draping over top of PT. |
| Flexor Digitorum Superficialis | Arm supine and elbow/forearm extended as much as possible.  Image approximately 50% down forearm. Important to see superficial and deep levels as demarcated as line between ulnar nerve and artery and fascia that connects to median nerve. |
| Pronator Teres | Arm supinated.  Upper 10-15% of forearm.  Radial aspect of supinated forearm.  The median nerve and radial artery are around the muscle.  The FCR is medial to pronator teres and has a wing over pronator teres. |
| **Lower limb muscles** |  |
| Rectus Femoris | Supine scanning at middle to upper thigh.  Muscle is superior to vastus intermedius and femur being most inferior.  Look for tendon within muscle belly for proper identification. |
| Medial Gastrocnemius | Prone scanning of upper calf approximately 10-15% distal from popliteal fossa.  Should see nice fusiform shape and trace muscle to isthmus. |
| Lateral Gastrocnemius | Prone scanning moving laterally from the isthmus approximately 10-15% distal from popliteal fossa. |
|  |  |

PT, Pronator teres; FCR, flexor carpi radialis

**Supplemental Table 2: Mean Muscle Strength (MRC 0-5) by Muscle Group and BoNT Exposure**

| Values are presented as Mean (Standard Deviation) for the affected side. | | |
| --- | --- | --- |
| **Muscle Group** | **BoNT Exposed Patients** | **BoNT Naive Patients** |
| Shoulder Abduction | 3.50 (1.63) | 3.50 (1.05) |
| Elbow Flexion | 3.54 (1.42) | 3.77 (1.18) |
| Grip (Hand) | 2.61 (1.69) | 2.50 (2.07) |
| Hip Flexion | 4.05 (1.09) | 3.50 (0.84) |
| Knee Extension | 4.36 (0.66) | 4.00 (1.10) |
| Ankle Dorsiflexion | 2.75 (1.76) | 3.00 (2.37) |
| MRC, Medical Research Council scale for muscle strength (0=paralysis, 5=normal); BoNT, botulinum neurotoxin | | |

|  |  |  |  |  |  |  |
| --- | --- | --- | --- | --- | --- | --- |
|  | | | | | | |

**Supplemental Table 3: Lifetime BoNT History by Muscle (Treated Muscles Only)**

| **BoNT Metric** | **Biceps brachii** | **Brachialis** | **Flexor carpi radialis** | **Pronator teres** | **Flexor digitorum superficialis** | | **Lateral gastrocnemius** | | | | **Medial gastrocnemius** | | | **Rectus femoris** |
| --- | --- | --- | --- | --- | --- | --- | --- | --- | --- | --- | --- | --- | --- | --- |
| **Cum. Dose (units)** | | |  |  |  |  | |  | |  |  |  |  |  |
| Median (Q1-Q3) | 368 (220-550) | 324 (260-510) | 260 (114-406) | 155 (98-345) | 580 (400-1,164) | | 240 (141-825) | | | | 684 (322-858) | | | 582 (293-815) |
| [Min-Max] | [104-1,390] | [30-1,050] | [30-1,146] | [20-1,368] | [76-2,630] | | [16-1,110] | | | | [60-1,600] | | | [202-850] |
| Not Reported, n (%) | 0 (0%) | 0 (0%) | 0 (0%) | 0 (0%) | 0 (0%) | | 0 (0%) | | | | 0 (0%) | | | 0 (0%) |
| **# BoNT Injection Cycles** | |  |  |  |  | | | |  | | |  |  |  |
| Median (Q1-Q3) | 7 (6-14) | 7 (5-11) | 8 (6-17) | 8 (4-13) | 14 (7-24) | | 6 (4-14) | | | | 10 (6-18) | | | 11 (10-20) |
| [Min-Max] | [3-33] | [1-16] | [1-45] | [1-44] | [5-41] | | [2-30] | | | | [1-32] | | | [9-27] |
| Not Reported, n (%) | 0 (0%) | 0 (0%) | 0 (0%) | 0 (0%) | 0 (0%) | | 0 (0%) | | | | 0 (0%) | | | 0 (0%) |
| **Days Since Last BoNT Injection** | | | |  |  |  | |  | |  |  |  |  |  |
| Median (Q1-Q3) | 469 (105-1,225) | 105 (105-119) | 123 (105-1,343) | 265 (112-1,574) | 105 (98-119) | | 112 (99-1,861) | | | | 119 (105-574) | | | 115 (102-905) |
| [Min-Max] | [93-3,281] | [93-1,482] | [98-3,281] | [98-3,281] | [93-434] | | [84-2,707] | | | | [84-2,714] | | | [98-1,685] |
| Not Reported, n (%) | 0 (0%) | 0 (0%) | 0 (0%) | 0 (0%) | 0 (0%) | | 0 (0%) | | | | 0 (0%) | | | 0 (0%) |
| **Avg. Dose/Cycle (units)** | | | |  |  |  | |  | |  |  |  |  |  |
| Median (Q1-Q3) | 52 (30-70) | 55 (27-66) | 28 (20-43) | 25 (15-32) | 61 (28-80) | | 42 (24-65) | | | | 62 (28-90) | | | 30 (26-59) |
| [Min-Max] | [17-79] | [16-75] | [7-58] | [13-50] | [8-188] | | [8-85] | | | | [12-107] | | | [22-85] |
| Not Reported, n (%) | 0 (0%) | 0 (0%) | 0 (0%) | 0 (0%) | 0 (0%) | | 0 (0%) | | | | 0 (0%) | | | 0 (0%) |

BoNT, botulinum neurotoxin; Cum., cumulative; Q1, first quartile; Q3, third quartile; Min, Minimum; Max, Maximum; n, number of patients; Avg., average
